# Response of microbiota to exogenous inoculation improved the enzymatic activities of medium-temperature *Daqu*

**DOI:** 10.3389/fmicb.2022.1047041

**Published:** 2022-11-15

**Authors:** Qianglin Pan, Jun Huang, Suyi Zhang, Hui Qin, Xiaojun Wang, Yu Mu, Huifang Tang, Rongqing Zhou

**Affiliations:** ^1^College of Biomass Science and Engineering, Sichuan University, Chengdu, China; ^2^Luzhou Lao Jiao Co., Ltd., Luzhou, China; ^3^National Engineering Research Center of Solid-State Manufacturing, Luzhou, China

**Keywords:** medium-temperature *Daqu*, functional isolates, enzymatic activity, sensitive, co-occurrence network

## Abstract

To explore the potential mechanism of improving enzymatic activities in medium-temperature *Daqu* (MTD) by inoculation functional isolates, we inoculated a single strain of *Bacillus licheniformis*, and the microbiota composed of *Bacillus velezensis* and *Bacillus subtilis* in MTD to investigate the association between the response of the functional microbiota and the enzymatic activity. The results showed that the bacterial community of MTD might be more sensitive to bioturbation than the fungal community, and the indigenous microbiota responded to the single strain more than to the microbiota. Moreover, the differential microorganisms mainly included *Lactobacillales*, *Bacillales*, and *Saccharomycetales* between the conventional and fortified samples. Notably, the composition of functional microbiota related to liquefying activity (LA) and saccharifying activity (SA) were significantly different, changing from *Lactobacillus* and *Rhizomucor* to *Bacillus*, *Weissella*, and *Hyphopichia*. That might be closely related to the effect of the bioturbation on LA (31.33%) and SA (43.54%) associated microorganisms was more tellingly. Furthermore, the relative abundance changes of bioturbation-sensitive modules in the co-occurrence network might also lead to the difference in enzymatic activities. Therefore, the LA and SA of MTD were improved by bioturbation significantly. These results provide diverse insights into the exogenous functional isolates to regulate the MTD microbiota and improve enzymatic activities.

## Introduction

*Daqu* is classified into three main types, namely high-, medium- and low-temperature *Daqu* (Zhou et al., 2022). They are not only an indispensable crude enzyme and starter but also one of the specific raw materials for Baijiu production (He et al., 2020; Xiao et al., 2021). Among them, the medium-temperature *Daqu* (MTD) is manufactured through spontaneous inoculation and fermentation, which allows its characteristics are endowed by the microbial community inhabiting the raw material and environment (Xiao et al., 2017; Du et al., 2019; Zhang et al., 2021).

In previous studies, the dominant microorganisms, metabolites, and their relationship in MTD have been preliminarily explored (Zheng et al., 2014; Li et al., 2020; Ma et al., 2021). It is a truism that the enzymatic activities of MTD closely lied to the microbial community structure and are manifested as a dynamic succession pattern (Liu et al., 2018; He et al., 2019; Guan et al., 2021). Yet, the succession was nonlinear during the process, which involved the interaction between the functional modules and the nutrition networks (Yi et al., 2019; Mao et al., 2022). The maximum information coefficient (MIC) analysis can capture linear and nonlinear relationships between independent and response variables (Reshef et al., 2011). This method could explore the relationships between microorganisms and environment variables and construct a microbial co-occurrence network (Logares et al., 2020; Lu et al., 2021). Perhaps, it also could explore the relationship between the trophic interaction, functional microbiota, and network modules to the community succession and enzymatic activities of MTD.

Moreover, the technology of inoculating functional isolates to improve the quality of MTD has become a hot topic. For example, the inoculation of *Bacillus licheniformis*, *Bacillus velezensis*, and *Bacillus subtilis* significantly improved the MTD enzymatic activities and flavor profile (Wang et al., 2017; He et al., 2019). Additionally, the content of ethyl caproate in the fermentation process of MTD was increased by inoculating *Saccharomyces cerevisiae*, and/or *Clavispora lusitaniae* (Li et al., 2020). However, the mechanism by which functional isolates drive the succession of indigenous communities and improve the function of MTD is still unclear. The latest works showed that the potential interactions among the different microbiota could be better understood by the co-occurrence network (Faust and Raes, 2012; Tang et al., 2021), and the exogenous species could change the co-occurrence pattern of indigenous microbiota (Carey et al., 2017; Yang et al., 2022). However, the critical role of exogenous strain/microbiota in regulating the network performance of indigenous communities in MTD was still uncertain.

In the present research, taking conventional and two types of fortified MTDs as the objects, the latter were inoculated with *B. licheniformis* and the microbiota composed of *B. velezensis* and *B. subtilis*, respectively, the response patterns of the functional microbiota of the MTD were explored. Then, MIC analysis was performed to determine the functional microbiota related to enzymatic activities and reveal the mechanism of the differences in enzymatic activities caused by different fortified patterns. These results will be beneficial for predicting the characteristics of fortified MTD and optimizing the manufacturing technique of MTD.

## Materials and methods

### Sources and functional characteristics of functional isolates

The *B. subtilis* D-31 and *B. velezensis* M-14 were isolated from conventional MTD at the famous Baijiu manufacturing enterprises located (Luzhou city and Yibin city, Sichuan Province, China). The *B. licheniformis* C-49 was isolated from the mutated MTD powder which stayed in space for a month and was loaded into the capsule of the Shenzhou 11 spacecraft. These isolates were identified by morphological and biochemical tests as well as 16S rDNA sequence (Fangio et al., 2020). Thereinto, the liquefying and saccharifying activities were significantly higher in MTD by inoculation with *B. subtilis* D-31 and *B. velezensis* M-14 (Xiao et al., 2014; Wu et al., 2017; He et al., 2019). Similarly, the enzymatic activities of MTD were also altered by inoculating *B. licheniformis* C-49 (Chen et al., 2021).

### Preparation of isolates suspension

Firstly, the activated isolates were inoculated into the commercial medium (LB, beef extract peptone medium) to prepare seed solution, respectively, and cultured at 37°C with shaking at 120 rpm/min for 24 h. Then, the 3 ml isolates suspension was transferred to 500 ml Aubergine flasks containing 100 ml agar slant of LB medium at 37°C for 24 h. Thirdly, the cultures were eluted with pre-prepared sterile water to prepare the suspension. Finally, the cell quantity of the suspension was counted by hemocytometer and diluted to 2.5 × 10^6^ CFU/ml with tap water.

### Experiment procedure and sampling

**Figure 1:**
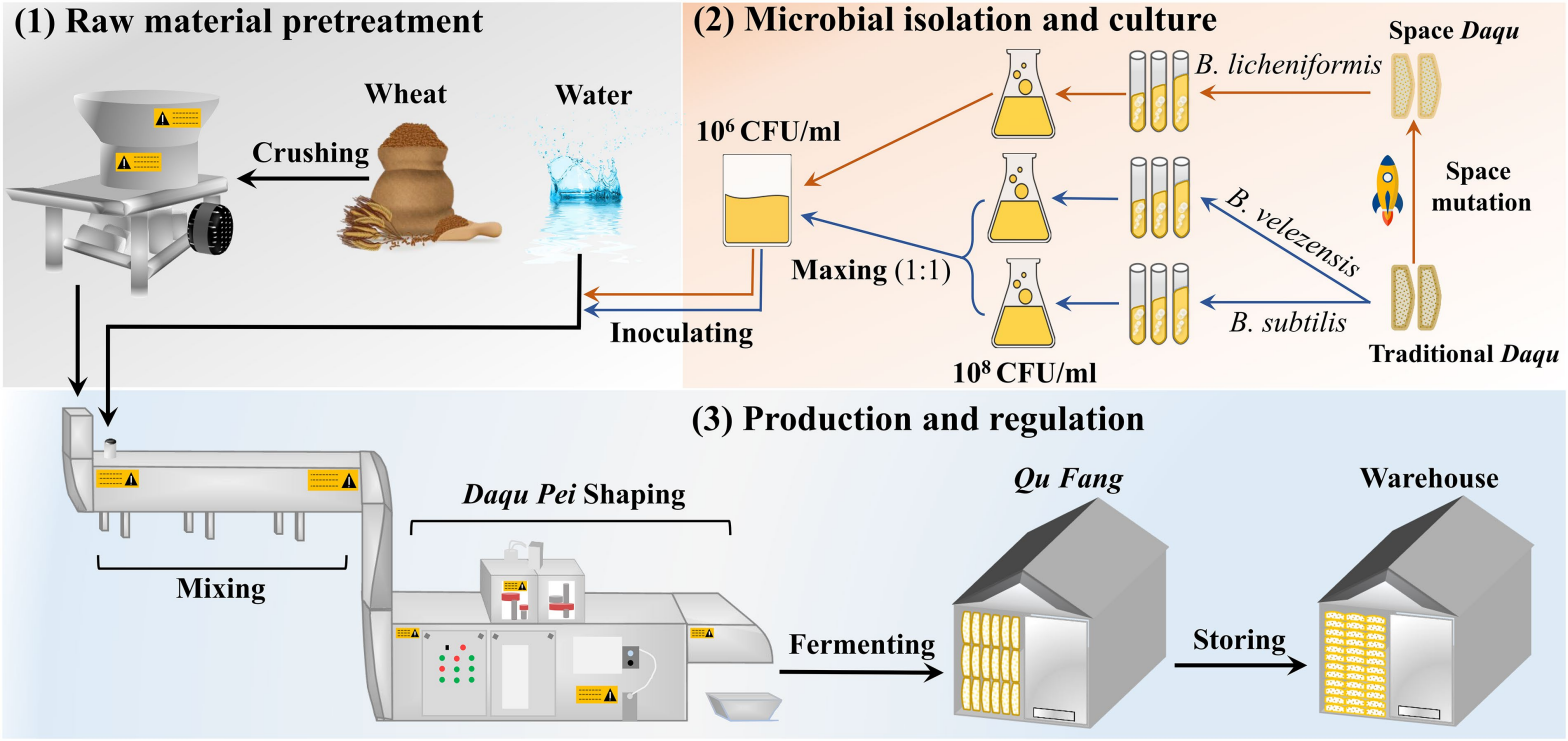
The manufacturing process of the medium-temperature *Daqu* (MTD).

The manufacturing process of the MTD is shown in Figure 1. Specifically, the crushed wheat was mixed with water containing the suspension (2.5 × 10^6^ CFU/ml) at a ratio of 0.62:0.38 and pressed firmly to shape a cuboid brick (*Daqu Pei*, 30 cm × 20 cm × 7 cm). Subsequently, the *Daqu* bricks were transferred and fermented in a fermentation room (called a *Qu Fang*) for about 12 days. Lastly, these bricks were stacked to another *Qu Fang* for aging to reach maturity. Among them, *Daqu Pei* was inoculated with the microbiota composed of *B. velezensis* and *B. subtilis* at a ratio of 1:1 abbreviated as DD, and with the single strain of *B. licheniformis* abbreviated as the CD. Uninoculated MTD was used as the blank and was called PD. The incubation of the MTD needs to be controlled strictly according to the following procedure. Firstly, the fermentation temperature of MTD increases up to 30–45°C within 2–3 days of the initial phase, gradually. And then, the temperature increases to a maximum of 58–61°C at a rate of 5–8°C/day. Finally, the temperature slowly decreased to below 35°C, indicating that MTD had then entered the mature process. During the entire fermentation process, the process parameters, such as the room temperature, humidity, and CO_2_ concentration, were regulated by opening and closing the doors and windows in time. Samples were collected separately on days 3, 4, 5, 6, 7, 10, 12, 15, 17, and 28 according to the previous method (He et al., 2019). And then, these samples were transported to our Lab *via* cold chain conveyance and stored at −20°C and −80°C, respectively, until analyzed. These samples were conducted biologically in triplicate.

### Physicochemical properties analysis

During the fermentation process, the temperature was measured in real-time by randomly inserting thermometers into *Daqu* bricks in each room. The moisture was measured by determining its weight loss after drying 5 g of MTD sample to a constant weight (105°C for 4 h). The content of total acidity was determined by acid–base titration to the endpoint of pH 8.2 with 0.1 mol/l NaOH solution. The liquefying, saccharifying, esterifying, and fermenting activities were detected by the previous method (Tang et al., 2019). All samples were measured in triplicate.

### Microbial community analysis

Total genomic DNA extraction was carried out using the Fast DNA SPIN extraction kits (MP Biomedicals, Santa Ana, CA, United States) according to the manufacturer’s instructions. For bacteria, the V3–V4 hypervariable region of the 16S rRNA gene was amplified by PCR with the universal primers of the forward 338F (5′-ACTCCTACGGGAGGCAGCA-3′) and the reverse 806R (5′-GGACTACHVGGGTWTCTAAT-3′). For fungi, the ITS1 was amplified by PCR with the primers of ITS5 (5′-GGAAGTAAAAGTCGTAACAAGG-3′) and ITS1 (5′- GCTGCGTTCTTCATCGATGC-3′). After the individual quantification, amplicons were pooled in equal quantities and subjected to high-throughput sequencing with a MiSeq Reagent Kit V3 (Personal, Shanghai, China) for pair-end 2 × 250 bp sequencing.

The sequences were analyzed with QIIME2 (2019.4), and the quality control of the sequences by using DADA2 (Callahan et al., 2016). Specifically, the default parameters of DADA2 were used for filtering and denoising. Secondly, the sequences were automatically calculated and spliced according to the clip length. Thirdly, the sequences with chimerism >8 were removed. Finally, the sequences with low abundance (reads ≤1) were removed. Each de-weighted sequence resulting from quality control using DADA2 is called ASV.

### Statistical and bioinformatics analyses

Unless otherwise stated, all statistical analyses were performed in R (v. 3.6.3). The dynamics of fermentation parameters, enzymatic activities, and microbial diversity were fitted *via* OriginPro2019 (OriginLab Corporation, MA, United States). Principal component analysis based on the Bray-Curtis distance was conducted by using the package “FactoMineR.” A one-way ANOVA and Stepwise multiple regression analyses were conducted using SPSS 25.0. The dissimilarity of the MTD microbial community and the correlation with fermentation parameters were assessed by principal coordinate analysis (PCoA) and Mantel tests, both of which were carried out in the “vegan” package. All possible Spearman’s rank correlations among α-diversity index, community ASVs and fermentation parameters using SPSS 25.0 and the significant correlations (|*ρ*| > 0.60, *p* < 0.05) were retained.

In this study, all co-occurrence networks were constructed in package “igraph.” Utilized the trimmed means of M (TMM) normalized counts per million (CPM) count, then conducted Spearman correlations among filtered ASVs with criteria the reads number >2 and prevalent in >4 samples, and retained the positive, significant correlations (*ρ* > 0.65, *p* < 0.001). To represent microbial groups, identified network modules within the networks (Li et al., 2021). The indicator species analysis was performed in the package “indicspecie” (De Cáceres et al., 2010), marked differential abundant ASVs using likelihood ratio tests (LRT) with FDR *p* < 0.05 in the package “edgeR” (Robinson et al., 2020). The MIC analysis was realized by using the package “minerva” (Reshef et al., 2011).

## Results

### Dynamics of fermentation parameters and enzymatic activities

**Figure 2:**
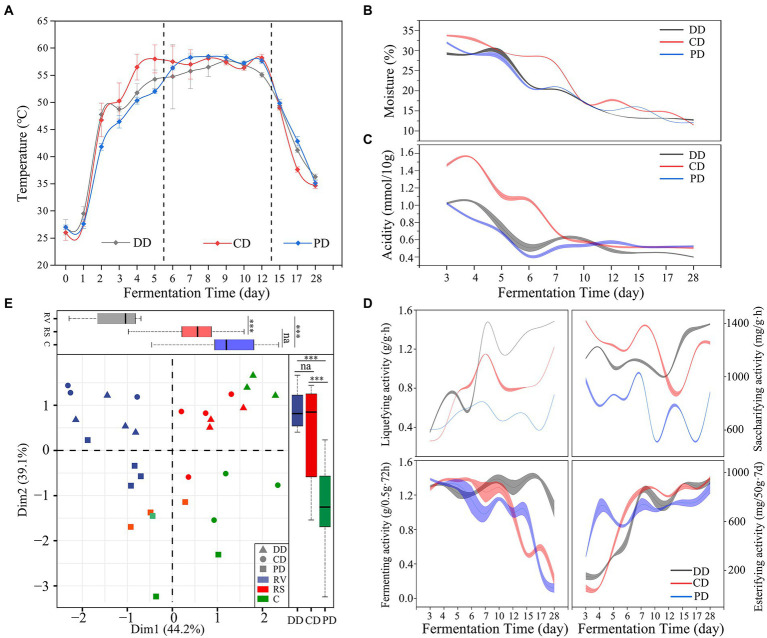
Fermentation parameters and enzymatic activity during the fermentation process. **(A–D)** The dynamics of fermentation parameters and enzymatic activity. **(E)** The phase (RV, days 0–6; RS, days 7–12; C, days 13–28) and type division according to principal component analysis based on the Bray–Curtis dissimilarity matrix of enzymatic activity (one-way ANOVA, ***p < 0.001).

As shown in Figure 2A, the dynamic temperature could be divided into three phases, which involved rising in vain (RV, days 0–6), the relative stability of high temperature (RS, days 7–12), and the cooling phase (C, days 13–28). In addition, the moisture and acidity were reduced markedly throughout the RV and RS phases, and in a slow and flat trend in the C phase (Figures 2B,C). The temperature changes of DD and CD were faster than that of sample PD in the RV phase (Figure 2A), while the moisture in the sample CD decreased the slowest (Figure 2B), which might be related to the spatial heterogeneity of solid fermentation and random sampling (Cai et al., 2022; Shi et al., 2022). Moreover, the trends of these parameters changing were similar in different samples (*p* > 0.05). In particular, the RS phase of all MTD samples was relatively stable.

The dynamics of enzymatic activities as shown in Figure 2D. These results suggested that the effect of inoculation functional isolates improved the activity of saccharifying and liquefying significantly (*p* < 0.001). Moreover, the enzymatic activities were different and separated from each other in the different groups of samples (Figure 2E), including the significant contribution of phase (PC1, 44.2%) and MTD type (PC2, 39.1%). These activities were notably related to the moisture for all samples and correlated with temperature and acidity for DD and CD based on multiple regression analyses (Supplementary Table S1).

### Microbial community structure and their succession during the process

**Figure 3:**
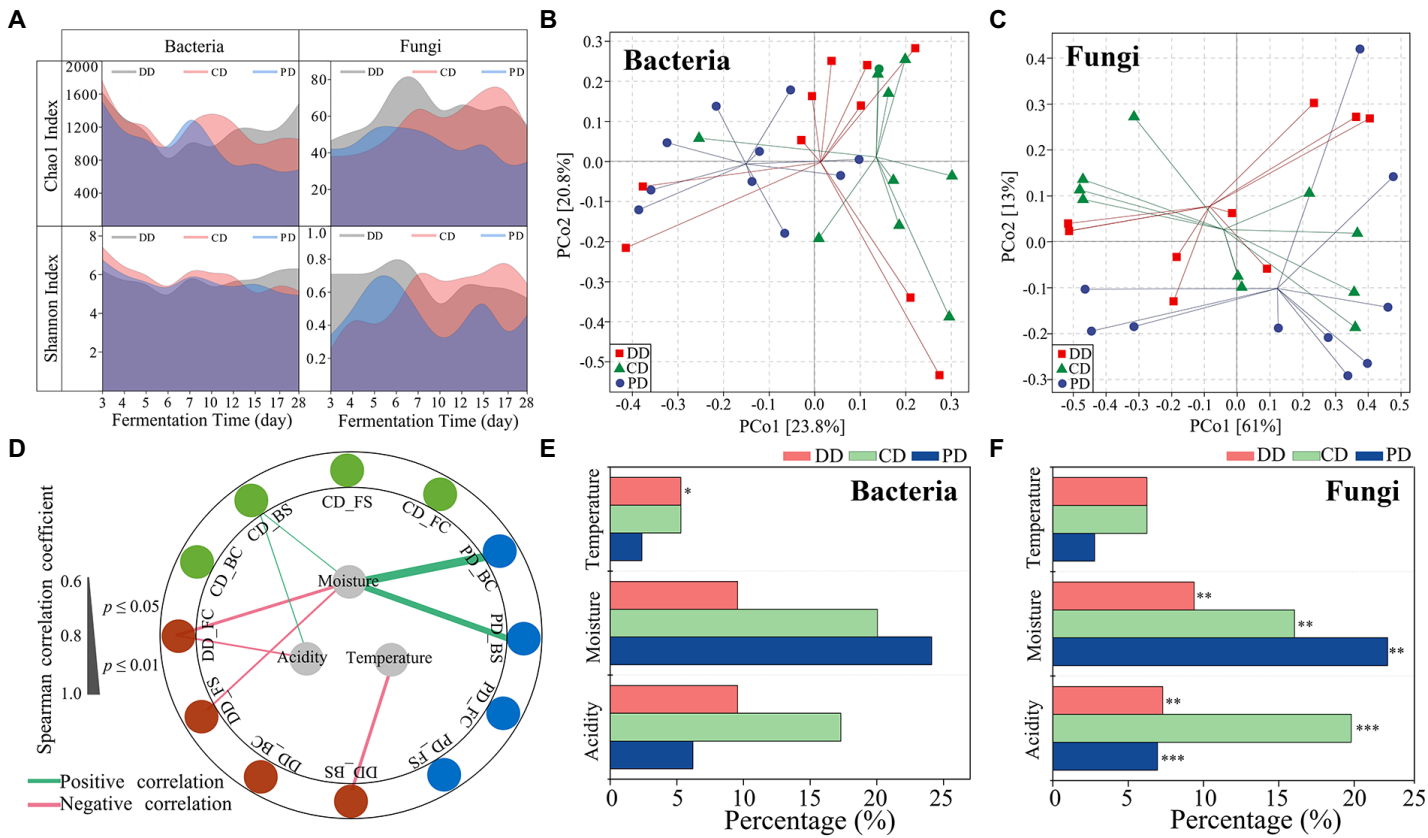
Microbial diversity and succession during the process. **(A)** Chao1 and Shannon indexes values for bacterial and fungal communities. **(B,C)** Group division according to principal coordinates analysis based on the Bray–Curtis dissimilarity matrix of microbial communities. **(D)** Correlation network between the α-diversity and fermentation parameters during the process (|*ρ*| > 0.60, *p* < 0.05). Abbreviations connected with underscores such as DD_BC, DD_BS indicated the bacterial Chao1 and Shannon index of the DD sample. **(E,F)** Percentage of the community turnover associated with different ecological processes as calculated using ASVs (Spearman, |*ρ*| > 0.60, *p* < 0.05). The significant effects of fermentation parameters on community are determined by mantel tests (***p < 0.001, **p < 0.01, *p < 0.05).

The effective sequences of bacteria and fungi were 63,806–102,077 and 75,339-103,151, respectively, and the high-quality sequences were 56,612–96,117 and 69,291-97,721, respectively. The proportions of high-quality sequences ranged from 88.73 to 94.16% for bacteria and 88.61 to 95.46% for fungi. The rarefaction curve indicated that the sequencing depth could characterize the diversity of the community (Supplementary Figures S1A,B). As shown in Figure 3A, the Chao1 and Shannon indices of bacteria and fungi were similar for the three types of MTD in the RV phase. However, the indices of community Chao1 and fungal Shannon in DD and CD were significantly higher than that of PD in the C phase (*p* < 0.05). The relationships between α-diversity and fermentation parameters as shown in Figure 3D. The results demonstrated that moisture played a major role in driving the success of α-diversity, which was significantly positively correlated with the bacterial α-diversity of PD, while negatively with the fungal community α-diversity of DD. In addition, the bacterial Shannon index of the DD was also negatively correlated with temperature.

The effect of the different fortification patterns on the microbial community structure in MTD was similar (Supplementary Figures S1C,D). The relative abundance (RA) of *Weissella* was always dominant, although it decreased during the fermentation process, whereas *Bacillus* gradually increased in all samples (Supplementary Figure S1C). In addition, the RA of *Lactobacillus* also increased during the C phase of DD, which was consistent with the results reported by He et al. (2019). The RA of *Pichia* remarkably decreased during the RS and C phases (Supplementary Figure S1D), the succession of *Rhizomucor* in PD was the same, while the *Thermomyces* was the opposite. Furthermore, the RA of *Rhizopus* and *Hyphopichia* in DD and CD dominated during the RV and RS phases rather than PD.

The results based on the PERMANOVA test showed that there were significant differences in the microbial community, including the bacteria (*R*^2^ = 0.109, *p* = 0.047), fungi (*R*^2^ = 0.453, *p* < 0.001) communities from the phases, and the bacterial community (*R*^2^ = 0.122, *p* = 0.032) from MTD types (Figures 3B,C; Supplementary Table S2). Moreover, the fungal communities of all samples were correlated with moisture and acidity by the Mantel test, while the bacteria community of sample DD was primarily related to fermentation temperature (Figures 3E,F; Supplementary Table S3). These results demonstrated that the process parameters play a vital role in community succession, and highlighted that it is necessary to reduce the interference of the process parameters when exploring the effects of functional isolates inoculation on MTD indigenous communities.

### Identifying bioturbation respond significantly microorganisms

The linear relationship between fermentation parameters and microbial species (ASVs) was characterize by Mantel test (*p* < 0.05) and Spearman rank correlation analysis (|*ρ*| > 0.60, *p* < 0.05). Defining these microorganisms as those primarily influenced by process parameters, the ASVs for bacteria and fungi were *bp*ASV and *fp*ASV, respectively (Figures 3E,F). The individual bacteria and fungi ASV whose RA varied among the different types of MTD were identified by the indicator species analysis and were named *bi*ASV and *fi*ASV, respectively (Supplementary Tables S4, S5). Notably, DD samples did not include specific *fi*ASV (Supplementary Table S5). As shown in Supplementary Figure S2, ASVs shared between *bp*ASVs and *bi*ASVs were highly limited in DD, while the ASVs between *fp*ASVs and *fi*ASVs were highly coincident in CD (45.45%) and PD (80%). It indicated that the significant difference in the bacterial community among the three types of MTD explained by the process parameters was indistinctive, whereas the differences in the fungal community were more likely to be explained. These results confirmed our previous analysis (Figures 3E,F; Supplementary Table S3).

**Figure 4:**
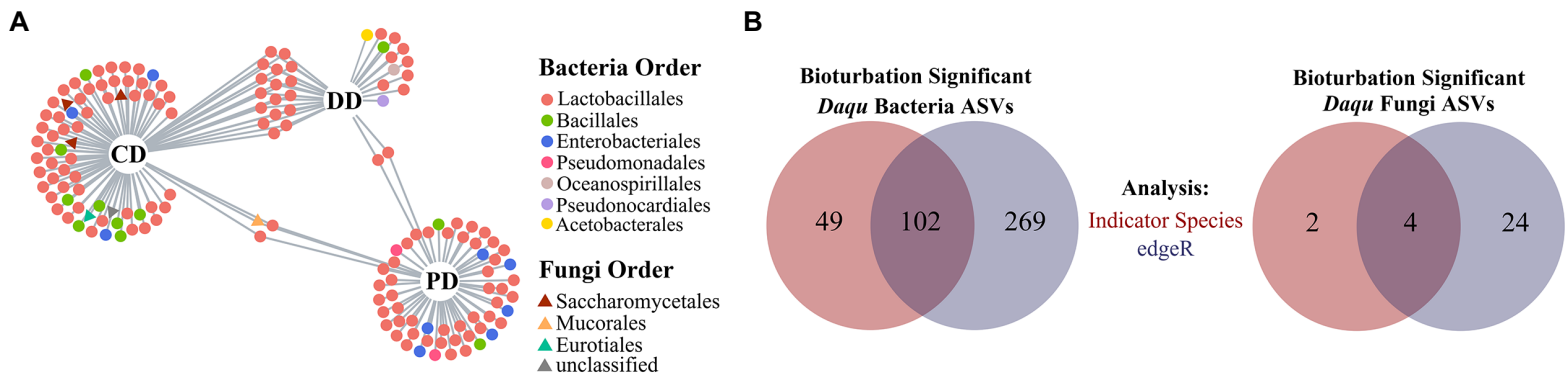
Defining the *bs*ASVs in which the MTD microbial communities respond significantly to bioturbation. **(A)** Bipartite networks display the distribution of differential ASVs (*br*ASVs). These ASVs are positively and significantly associated (*p* < 0.05) with one or more of the MTD types. **(B)** Venn diagrams show the number of ASVs significantly responding to bioturbation identified with specific ASVs of bioturbation (red) and by edgeR (blue). These ASVs are defined as bioturbation responding significantly ASVs (*bs*ASVs).

As mentioned above, the ASVs driven by bioturbation effects might also be influenced by process parameters. Therefore, the remaining *i*ASVs thought removing the *p*ASVs were defined as *br*ASV that were affected by bioturbation, and 151 bacterial and six fungal *br*ASVs were obtained (Supplementary Tables S4, S5). Concretely, bacterial *br*ASVs were mainly composed of *Lactobacillales*, *Bacillales*, and *Enterobacteriales*. While the fungal *br*ASVs were composed of *Saccharomycetales* and prominent associated with the CD (Figure 4A). In addition, there were abundant *br*ASVs in three types of MTD, and various bacterial *br*ASVs were shared between DD and CD (Supplementary Table S4; Figure 4A), which reflected the cluster of these samples in the ordination (Supplementary Table S2). Further, it was defined as *br*ASVs that were supported by the likelihood ratio test (FDR, *p* < 0.05) as bioturbation responding significant ASVs (hereafter: *bs*ASVs), and a total of 102 bacterial (DD: 19, CD: 52, PD: 41) and four fungal *bs*ASVs (CD: 4) were identified (Figure 4B; Supplementary Tables S4, S5). These results showed that the indigenous microbial community of MTD was more responsive to the bioturbation of *B. licheniformis* than to the microbiota composed of *B. velezensis* and *B. subtilis*. As an approximation for the “effect size” of bioturbation on microbial communities by Hartman et al. (2018), these *bs*ASVs accounted for 11.95 and 3.67% of the total bacterial and fungal sequences, respectively, suggesting that the bacterial community of MTD might be more sensitive to bioturbation.

### Bioturbation effects on microbial co-occurrence patterns

**Figure 5:**
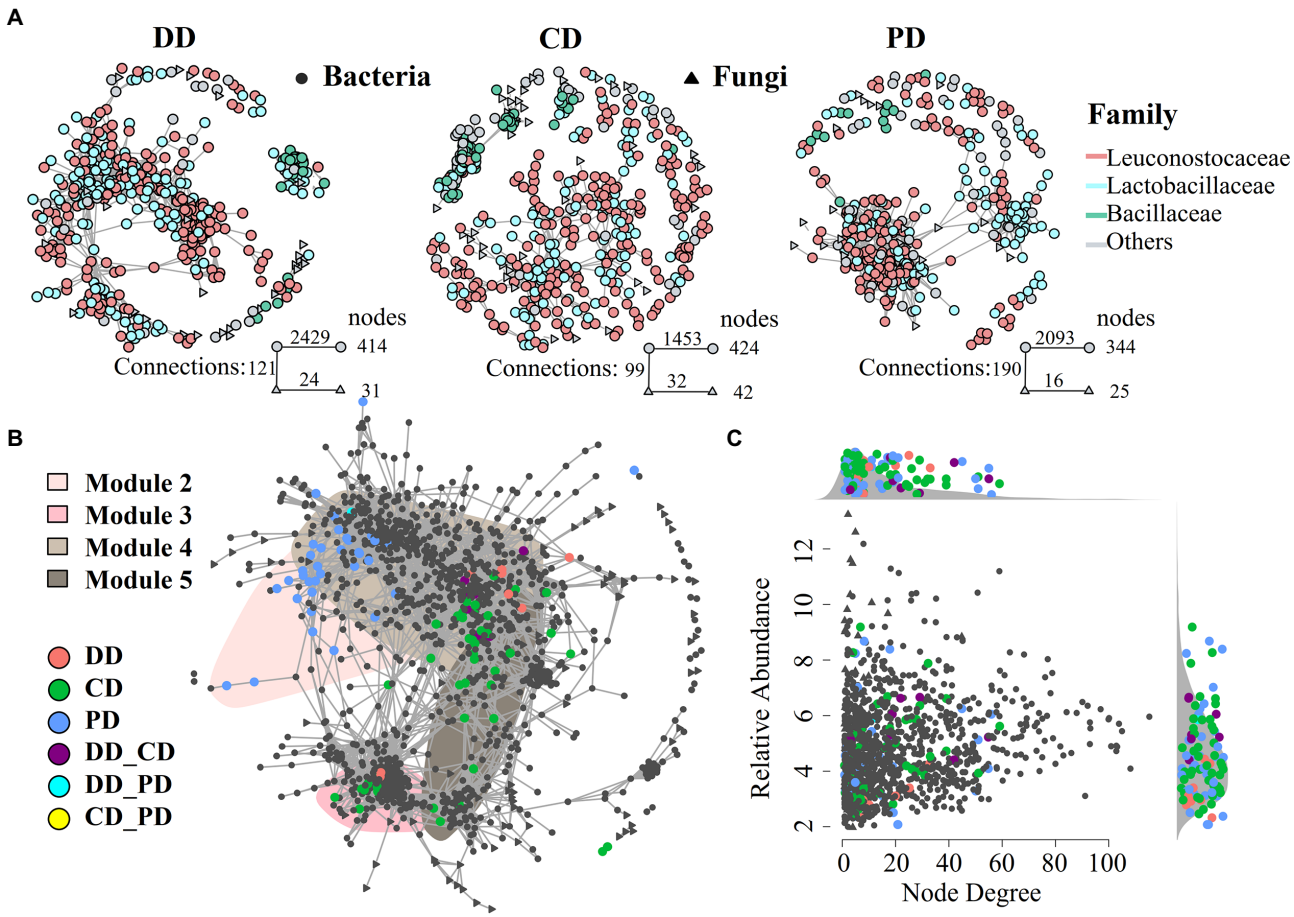
The MTD microbial community co-occurrence patterns. **(A)** Co-occurrence network of different types MTD microbial communities (*ρ* > 0.65, *p* < 0.001). **(B)** Full sample co-occurrence network and distribution of bioturbation responding significant ASVs (*bs*ASV) under specific bioturbation, insensitive ASVs were colored in gray. The major network modules containing *bs*ASVs were shaded. Abbreviations connected with underscores such as DD_CD, DD_PD, CD_PD indicated the joint *bs*ASVs of different MTD. **(C)** Degree of co-occurrence and abundance of *bs*ASVs. Relative abundance (as counts per million, CPM) of all ASVs from the MTD microbiome co-occurrence networks is plotted as a function of their degree of co-occurrence. Side panels recapitulate the distributions of co-occurrence degrees and abundance for the *bs*ASVs compared to the density of all.

To further investigate the characteristics of indigenous microbiota in response to bioturbation by network analysis. As shown in Figure 5A, networks of fortified MTD (DD and CD) differed from conventional MTD (PD), and the former had a larger network size (more nodes), which was consistent with an increase in species richness of fortified MTD (Figure 3A). Among them, the DD network was more connected (larger number of links), while the CD network contained more modules. A total of 19, 29, and 27 modules were identified in samples DD, CD, and PD, respectively, suggesting that the response of indigenous microbiota in MTD varied with the fortified pattern.

As shown in Figures 5B, a robust and suitable co-occurrence network was constructed based on the microbiota of all samples (Hartman et al., 2018; Li et al., 2021), and the *bs*ASVs that were closely associated with the core modules were identified. These results showed that the bioturbation changed the co-occurrence network and the RA of core modules (Figure 5B; Supplementary Figure S3). Among the top 10 modules, four modules (M2, M3, M4, and M5) contained abundant nodes of *bs*ASVs and were defined as bioturbation-sensitive modules (hereafter: *bs*Modules), while other modules were the insensitive modules. The distribution of these *bs*Modules in the network reflected the community dissimilarity in PCoA ordinations (Figures 3B,C). For instance, the *bs*ASV nodes of the PD were distributed in M2 and separated from other modules, while the modules of M3 and M4 have abundant *bs*ASV nodes of samples DD and CD, so they were closely connected. Equally, the RA of *bs*Module in different samples had a similar trend, while the modules of M2 and M3 involved in samples CD and DD contained most of *Hyphopichia* and *Bacillus*, respectively (Supplementary Figures S1C,D, S3A). In addition, the RA of all ASVs from the co-occurrence networks was plotted as a function of their degree of co-occurrence, abundant *bs*ASVs were identified among low node degree and lower RA microbiota (Figure 5C).

### Associating enzymatic properties with microbial community

**Figure 6:**
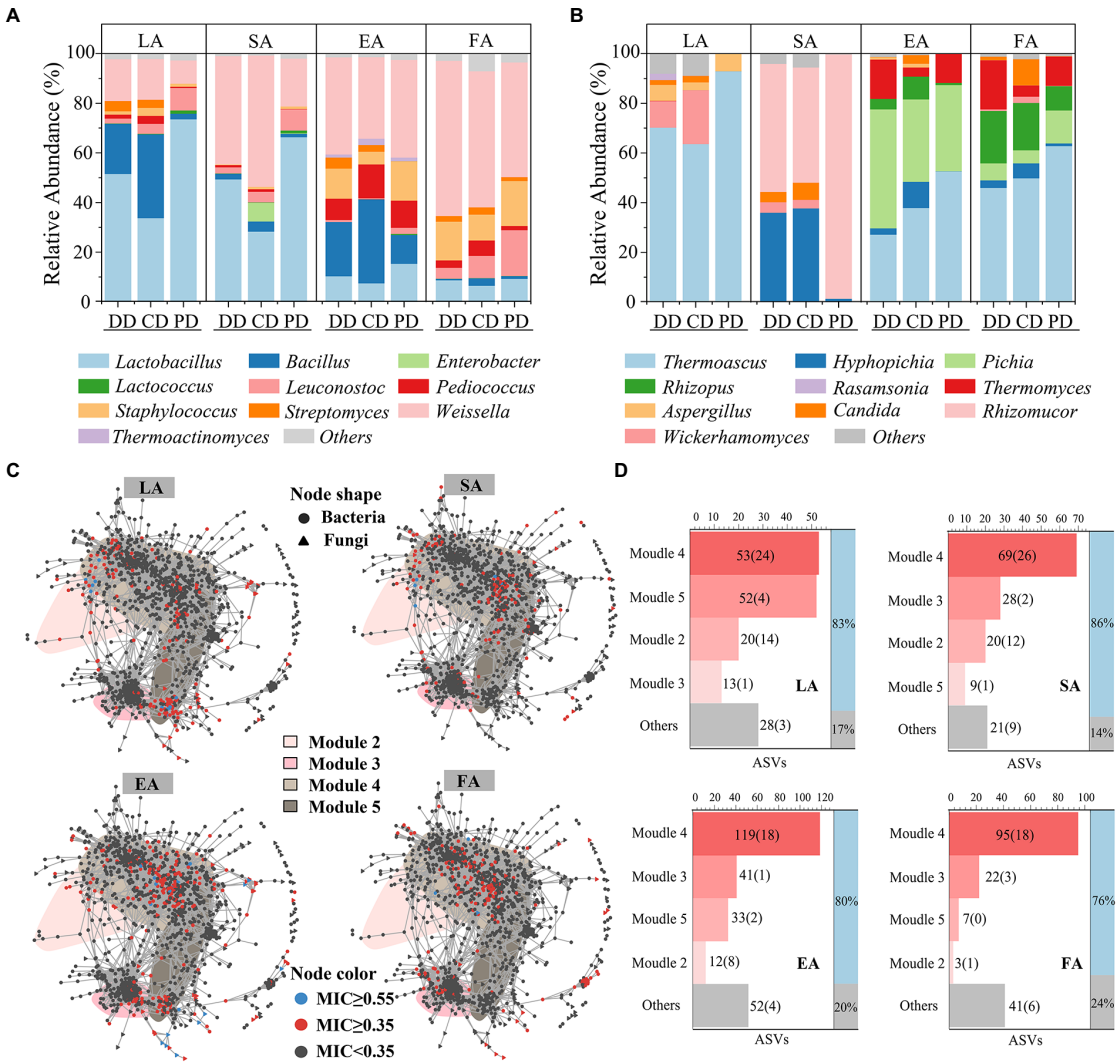
Correlation analysis *Daqu* microbial community with enzymatic activity. Community composition of bacterial **(A)** and fungal **(B)** that contribute significantly (*e*ASVs, maximum information coefficient ≥0.35) to MTD enzymatic activity, expressed by the average relative abundance of all samples of each type MTD during the fermentation process. **(C)** Distribution of *e*ASVs in co-occurrence network. LA, SA, EA and FA, respectively, represent liquefying-, saccharifying-, esterifying- and fermenting-activity. **(D)** The number of *e*ASVs (*bs*ASVs) in *bs*Module, “Others” represents the total number of *e*ASVs distributed in insensitive modules. The ratio of *e*ASVs contained in *bs*Module to all detected *e*ASVs is shown in the right histogram.

The potential links identified between enzymatic activities and ASVs by MIC analysis (MIC ≥0.35; Reshef et al., 2011), 166, 147, 257, and 168 ASVs were strongly associated with LA, SA, EA, and FA, respectively, and they were defined as *e*ASVs. These *e*ASVs qualitative taxonomic composition by per type sample genus average RA. As shown in Figures 6A,B, the dominant functional microbiota related to enzymatic activities included *Lactobacillus*, *Weissella*, *Bacillus*, *Hyphopicha*, and *Pichia*, which was in agreement with previous studies (Wang et al., 2017; He et al., 2019). Among them, the composition of LA- and SA-related fungal and LA-related bacterial functional microbiota in fortified MTD was significantly different from that of PD (Supplementary Table S6). Specifically, the average RA of *Bacillus*, *Weissella*, and *Wickerhamomyces* were strongly correlated with LA, and that of *Hyphopicha* with SA. These genera were significantly increased in the fortified MTD, while *Lactobacillus* and *Rhizomucor* were the opposite (Supplementary Table S7).

As shown in Figure 6C, the *e*ASVs nodes aggregation in the co-occurrence network. Thereinto, the distributions of *e*ASVs related to LA and SA were similar and highly overlapped with the *bs*ASVs, and the *e*ASVs with MIC ≥0.55 (Chen et al., 2020; Logares et al., 2020) distributed in M4 and M2 were primarily. The distributions of *e*ASVs related to EA and FA in the non-*bs*ASV area of M4 were also similar. The statistical results of *e*ASVs attributes in the network showed that the proportion distributed by the four *bs*Modules was as high as 76–86% (Figure 6D), which implied the change of *bs*Modules significantly affected the enzymatic activities of MTD. Next, we used the ratio of *bs*ASVs in *e*ASVs to characterize the effect intensity of bioturbation on the MTD enzymatic activities (Supplementary Figure S3E). The response of different enzymatic activities to bioturbation was diverse, with the impact on SA (43.54%) being the largest, followed by LA (31.33%) and EA (15.56%). These results revealed the diverse enzymatic activities among different MTD samples.

## Discussion

Previous works have proven that temperature, acidity, and moisture could affect the microbial composition of MTD (Deng et al., 2021; Ma et al., 2021). The exogenous inoculation of functional isolates accelerated the metabolism of the indigenous community in the early phase and increased the temperature and acidity rapidly (Figures 2A,C), thereby significantly changing the community structure and enhancing the effect on enzymatic activities (Supplementary Figures S1C,D). Meanwhile, the higher temperature accelerated the decrease in moisture, resulting in the loss of some species and genera that were difficult to survive under low moisture, including *Pichia*, *Rhizopus*, and *Hyphopichia*. Conversely, this change promoted the enrichment of thermophilic microorganisms, namely *Bacillus*, *Thermoascus*, and *Thermomyces*. These results also led to the variation in enzymatic activities (Figure 2D). However, the evolutionary trends of the process parameters were similar across groups, which only partially explained the difference in microbiota between the fortified and conventional MTD. Inoculating function isolates significantly changed the community diversity and structure (He et al., 2019; Li et al., 2020), emphasizing the importance of selecting functional isolates and optimizing fortifying patterns.

The response of the indigenous community in MTD showed partial similarity to different fortifying patterns, while it was more responsive to the inoculation of *B. licheniformis* (Figure 4A; Supplementary Tables S3–S5). In addition, the bacterial community in MTD was more sensitive to bioturbation than the fungal community because *bs*ASVs mainly belonged to bacterial microbiota, which was contrary to the patterns found by Wang et al. (2017). These results indicate that the MTD community might display different responding mechanisms for inoculating different functional isolates. The RA of *bs*ASV in the community of MTD was smaller, and most genera were more likely to be classified as rare rather than abundant taxa (Figure 5C). Previous studies suggested that the rare taxa were more prone to migrating rather than resisting changes (Shade et al., 2014; Jiao et al., 2019), and contain abundant metabolically active lineages (Dohrmann et al., 2013; Xiong et al., 2021). Thus, they might respond to bioturbation rapidly, resulting in changes in composition and abundance of the microbial community to adapt to external stresses for better survival (Li et al., 2021).

The co-occurrence network has been widely used to explore potential correlations among the communities (Faust and Raes, 2012; Tang et al., 2021). It contributed to exploring how the inoculated functional isolates affect the ecological pattern of indigenous communities in MTD (Yang et al., 2022). In the present study, we found that the bioturbation of the functional isolates could result in a significant change in the abundance of the dominant microorganism in MTD. In addition, the microbiota closely associated with variation in enzymatic activities was also changed (Supplementary Figure S3A). For example, increased *Bacillus* might lead to LA increase, while the improvement of SA may be attributed to the enrichment of *Hyphopichia* (Kurtzman, 2011). The aggregation of *e*ASVs related to the identical enzymatic activity in the co-occurrence network indicated that microorganisms with similar functions cooperated closely because they had niche breadths similarly (Hartman et al., 2018; Li et al., 2021). That also explained the reason by inoculation of the *Bacillus* isolates significantly increased LA and SA in MTD.

The external disturbance could affect interspecies interactions by changing the “accessory microbiome” and embodied in different nutrition patterns (Hartman et al., 2018; Li et al., 2021), which might cause the change of MTD enzymatic activities related to the abundance of *bs*Moudles. For example, the *e*ASVs of LA by bioturbation were mainly distributed in M2 and M4 (Figure 6D), and the average abundance of these *bs*Modules in the RV phase of the DD and CD was higher than that of the PD (Supplementary Figure S3E). Meanwhile, this phase was the main period of enzyme production and accumulation (Li et al., 2017). Therefore, the increased RA of functional microbiota was conducive to accelerating the accumulation of enzymatic activity, thereby enhancing the LA, SA, EA, and FA. These results showed that the changes in the abundance of identified *bs*Modules could partially explain the differences in the enzymatic activities across MTD groups, and also suggested that the *bs*ASVs could promote the variation of accessory ASVs.

## Conclusion

This study revealed the effects of exogenous inoculation on the microbial community and enzymatic activities of MTD by high-throughput sequencing and ecological statistic method. The results suggested that the enzymatic activities of MTD were closely related to the response patterns of functional microbiota and network modules to inoculation. Briefly, the microbiota related to LA and SA were changed from *Lactobacillus* and *Rhizomucor* to *Bacillus*, *Weissella*, and *Hyphopichia*. Meanwhile, the abundance patterns of *bs*Modules were affected. Ultimately, these changes promote improved enzymatic activity in MTD, especially LA and SA. This work is beneficial for controlling MTD quality by regulating microbiota assembly and provides theoretical insights into the regulation mechanism.

## Data availability statement

The datasets presented in this study can be found in online repositories. The names of the repository/repositories and accession number(s) can be found at: https://www.ncbi.nlm.nih.gov/, PRJNA807119.

## Author contributions

QP performed the experiments, analyzed the data and prepared the manuscript. JH guided the experiments. SZ, HQ, and XW provided financial support and sample collection. YM and HT contributed to data curation and manuscript revision. RZ contributed to the experimental design, manuscript revision, and overall support of this study. All authors contributed to the article and approved the submitted version.

## Funding

This research was funded by the Cooperation Project of Luzhou Lao Jiao Co., Ltd. and Sichuan University (no. 21H0997).

## Conflict of interest

SZ, HQ, XW, and RZ were employed by Luzhou Lao Jiao Co., Ltd.

The remaining authors declare that the research was conducted in the absence of any commercial or financial relationships that could be construed as a potential conflict of interest. The authors declare that this study received funding from Luzhou Lao Jiao Co., Ltd. The funder had the following involvement in the study: raw materials and site providing, sample collection, and decision to publish.

## Publisher’s note

All claims expressed in this article are solely those of the authors and do not necessarily represent those of their affiliated organizations, or those of the publisher, the editors and the reviewers. Any product that may be evaluated in this article, or claim that may be made by its manufacturer, is not guaranteed or endorsed by the publisher.
