# Supplementary material for: Response of microbiota to exogenous inoculation improved the enzymatic activities of medium-temperature Daqu
Source: Front Microbiol. 2022 Nov 15;13:1047041. doi: 10.3389/fmicb.2022.1047041 (PMC9706721; doi:10.3389/fmicb.2022.1047041)
Supplement: Supplementary file 1 [file Data_Sheet_1.docx]

Supplementary Material

# Supplementary Figures and Tables

## Supplementary Figures


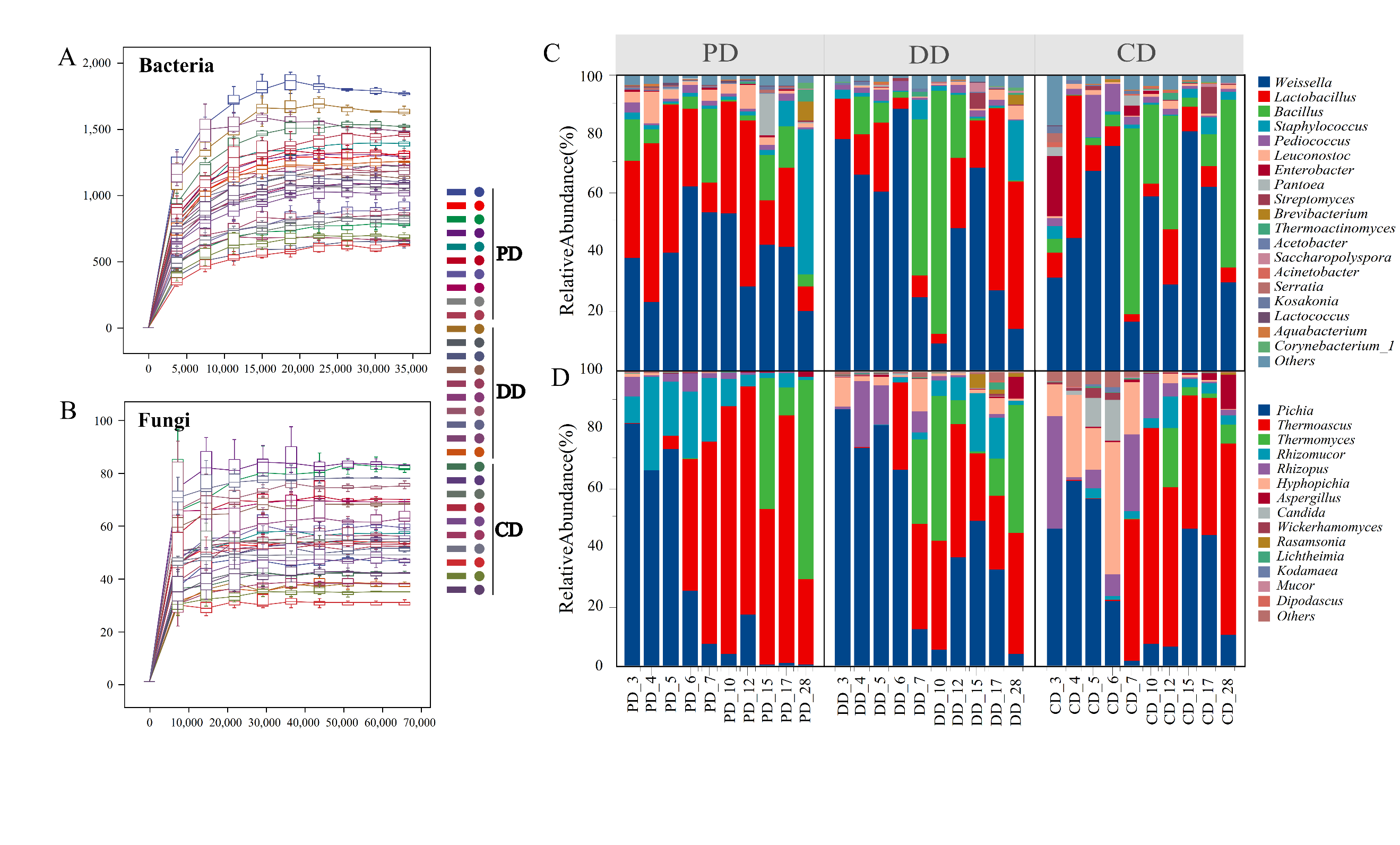


**Supplementary Figure 1.** **(A-B)** The sparse curve of the microbial community; **(C-D)** Relative abundance of the microbial community. Only those genera that had an average abundance greater than 1% in at least one sample are indicated. Genera with less than 1% abundance are combined and shown in the “Others” category.


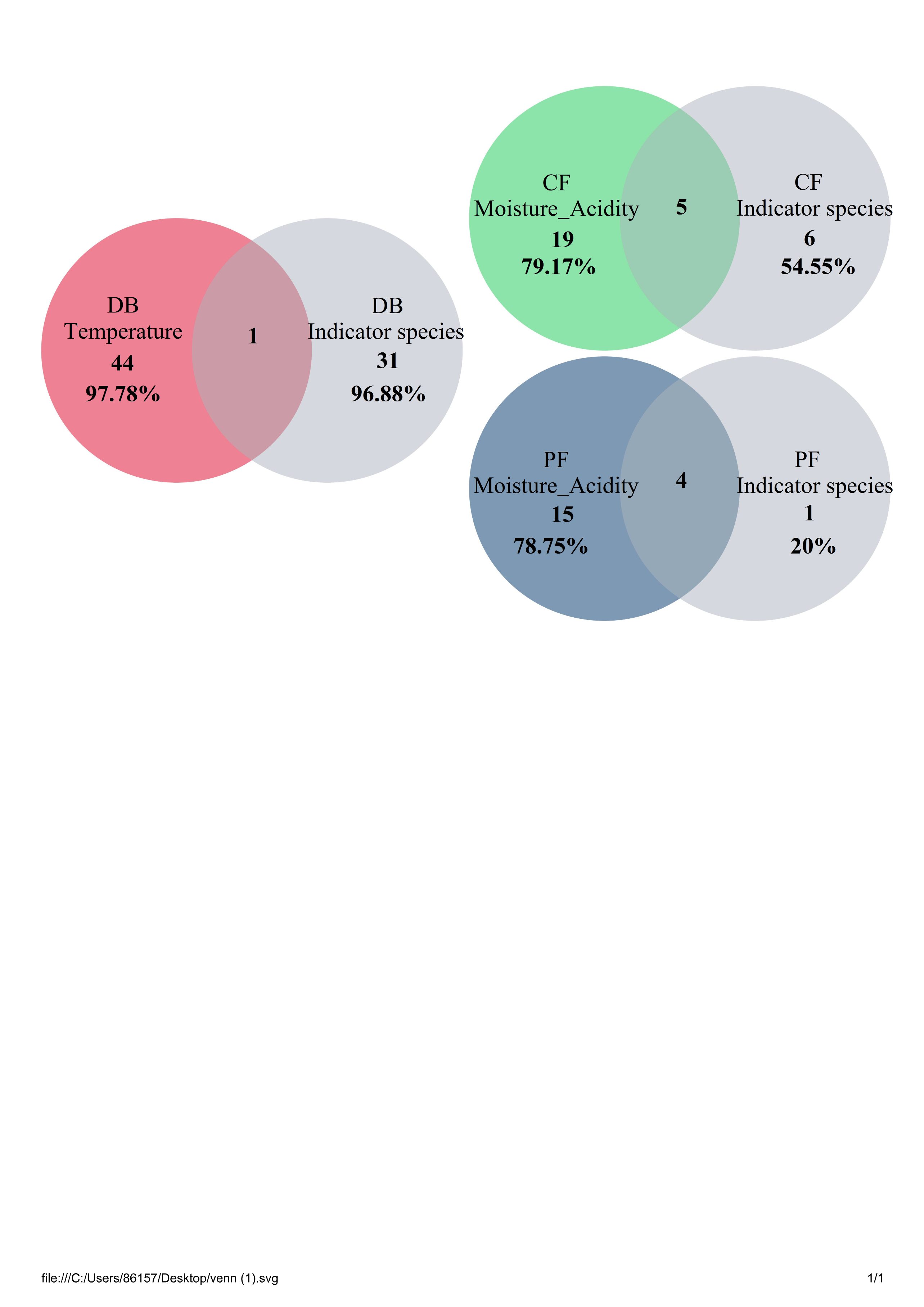


**Supplementary Figure 2.** Defining the response ASVs of the MTD community to bioturbation. First, determine the ASVs that are mainly driven by fermentation parameters (for example, DB Temperature means the bacterial ASVs in the DD group that are mainly driven by fermentation temperature); Then, in the analysis results of the indicator species, the differential ASVs dominated by fermentation parameters are removed, and this is defined as the response ASVs of the MTD microbial community to bioturbation.


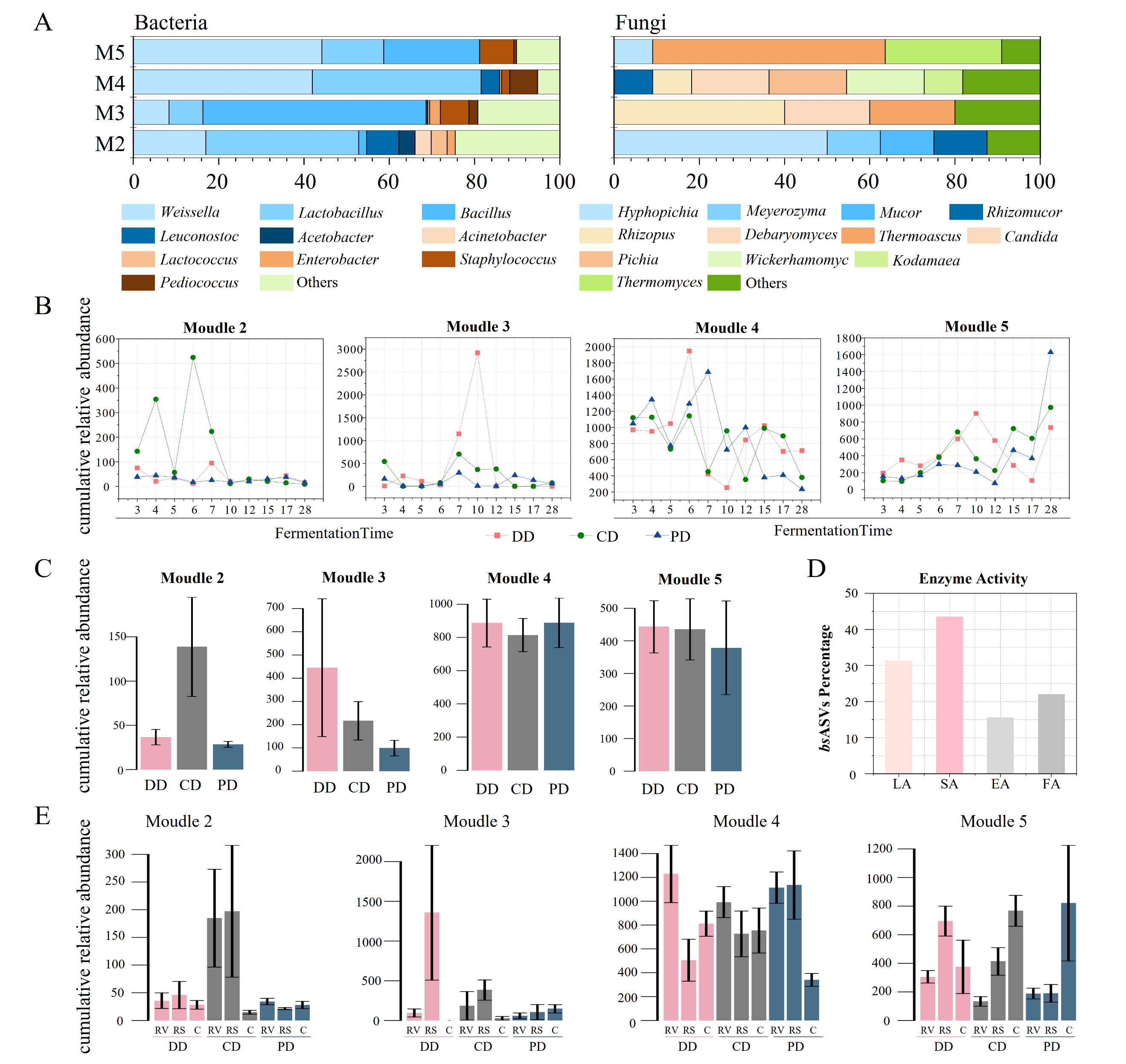


**Supplementary Figure 3.** **(A)** Qualitative taxonomic composition of *bs*Modules is reported as proportional ASVs numbers per genus; **(B-C and E)** Relative abundance (as counts per million, CPM; y-axis in ×1000) of all bacteria and fungi of the *bs*Modules in networks (RV, days 0 to 6; RS, days 7 to 12; C, days 13 to 28); **(D)** The proportion of different enzyme activity corresponding to *bs*ASVs contained in *e*ASVs **(Figure 5D)**.

## Supplementary Tables

**Supplementary Table 1** Regression analyses of MTD enzyme activity composition with fermentation parameters.

| **Groups** | **Model r^2^** | **Model Sig.** | **Explanatory variables** |
| --- | --- | --- | --- |
| DD | 0.811^a^，0.367^b^ | 0.000^a^，0.037^b^ | Moisture^a***^，T^b*^ |
| CD | 0.827^a^, 0.700^b^ | 0.000^a^，0.002^b^ | Acidity^a***^，Moisture^b**^ |
| PD | 0.424^a^, 0.721^b^ | 0.025^a^，0.001^b^ | Moisture^a**^，Moisture^b**^ |

**Note:** Multiple regression analyses of enzyme activity composition were performed as a function of fermentation parameters. For enzyme activity composition, the first and second axis of PCA scaling was used as the response variables. Regression method adopts Stepwise, the probability *P* ≤ 0.05 was included in the explanatory variable, and *P* ≥ 0.10 was the excluded variable.

a: first PCA axis; b: second PCA axis.

* *P* < 0.05, ** *P* < 0.01, *** *P* < 0.001.

**Supplementary Table 2** Results of PERMANOVA testing of different types of MTD microbial communities.

|  | **group** | **distance** | **Variation (R2)** | **Pr (>F)** | **P_adj_BH** |
| --- | --- | --- | --- | --- | --- |
| **Bacteria** | CD/DD | Bray-Curtis | 0.054711 | 0.384 | 0.384 |
|  | CD/PD | Bray-Curtis | 0.158579 | **0.003** | **0.009** |
|  | DD/PD | Bray-Curtis | 0.072724 | 0.186 | 0.279 |
| **Fungi** | CD/DD | Bray-Curtis | 0.07525 | 0.22 | 0.22 |
|  | CD/PD | Bray-Curtis | 0.094857 | 0.114 | 0.2145 |
|  | DD/PD | Bray-Curtis | 0.099163 | 0.131 | 0.2145 |

**Supplementary Table 3** Results of Mantel testing between MTD microbial community (ASVs) and fermentation parameters.

| Env | DD | | | | CD | | | | PD | | | |
| --- | --- | --- | --- | --- | --- | --- | --- | --- | --- | --- | --- | --- |
|  | Bacteria | | Fungi | | Bacteria | | Fungi | | Bacteria | | Fungi | |
|  | *rM* | *P* | *rM* | *P* | *rM* | *P* | *rM* | *P* | *rM* | *P* | *rM* | *P* |
| Temperature | **0.5423** | **0.0192** | 0.1235 | 0.1774 | 0.02095 | 0.3992 | 0.0562 | 0.2809 | 0.1816 | 0.1717 | 0.1576 | 0.1669 |
| Acidity | —— | —— | **0.4479** | **0.01** | 0.1614 | 0.1638 | **0.7812** | **7.00E-04** | —— | —— | **0.6115** | **0.0051** |
| Moisture | —— | —— | **0.6264** | **0.0037** | 0.1202 | 0.2072 | **0.6679** | **0.0011** | 0.1082 | 0.2299 | **0.8252** | **3.00E-04** |
| Total | 0.1096 | 0.2665 | **0.4453** | **0.0096** | 0.1393 | 0.1969 | **0.6014** | **7.00E-04** | 0.2242 | 0.1563 | **0.7393** | **3.00E-04** |

**Note:** Mantel statistics by *rM*, the significance by *P*, and Total was the test of all fermentation parameters. Missing values in the table indicate that the test results were negative.

**Supplementary Table 4** Definition of bacterial ASV (*b*ASV) sensitive to bioturbation.

| **Group** | **DD** | **CD** | **PD** | **Genus** |
| --- | --- | --- | --- | --- |
| **bASV_16199** | **1** | **0** | **1** | ***Gluconobacter*** |
| **bASV_1205** | **1** | **0** | **1** | ***Lactobacillus*** |
| bASV_1345 | 1 | 0 | 0 | *Frateuria* |
| bASV_5136 | 1 | 0 | 0 | *Carnimonas* |
| **bASV_23036** | **1** | **0** | **0** | ***Weissella*** |
| **bASV_23516** | **1** | **0** | **0** | ***Lactobacillus*** |
| **bASV_14726** | **1** | **0** | **0** | ***Saccharopolyspora*** |
| bASV_5323 | 1 | 0 | 0 | *Weissella* |
| **bASV_18510** | **1** | **0** | **0** | ***Lactobacillus*** |
| **bASV_455** | **1** | **0** | **0** | ***Lactobacillus*** |
| **bASV_6956** | **1** | **0** | **0** | ***Bacillus*** |
| **bASV_8792** | **1** | **0** | **0** | ***Weissella*** |
| **bASV_9357** | **1** | **0** | **0** | ***Lactobacillus*** |
| **bASV_11208** | **1** | **0** | **0** | ***Weissella*** |
| bASV_5192 | 1 | 1 | 0 | *Weissella* |
| bASV_22692 | 1 | 1 | 0 | *Weissella* |
| bASV_1402 | 1 | 1 | 0 | *Weissella* |
| bASV_12727 | 1 | 1 | 0 | *Weissella* |
| bASV_11481 | 1 | 1 | 0 | *Weissella* |
| **bASV_7671** | **1** | **1** | **0** | ***Lactobacillus*** |
| bASV_3367 | 1 | 1 | 0 | *Weissella* |
| **bASV_3544** | **1** | **1** | **0** | ***Weissella*** |
| bASV_4474 | 1 | 1 | 0 | *Weissella* |
| bASV_22390 | 1 | 1 | 0 | *Weissella* |
| **bASV_3909** | **1** | **1** | **0** | ***Weissella*** |
| bASV_18571 | 1 | 1 | 0 | *Weissella* |
| **bASV_10891** | **1** | **1** | **0** | ***Weissella*** |
| **bASV_11206** | **1** | **1** | **0** | ***Weissella*** |
| **bASV_17087** | **1** | **1** | **0** | ***Pediococcus*** |
| **bASV_15287** | **1** | **1** | **0** | ***Weissella*** |
| bASV_18504 | 1 | 1 | 0 | *Weissella* |
| bASV_12730 | 1 | 0 | 1 | *Weissella* |
| **bASV_867** | **1** | **1** | **0** | ***Bacillus*** |
| bASV_5372 | 0 | 1 | 0 | *Weissella* |
| bASV_4683 | 0 | 1 | 0 | *Pediococcus* |
| bASV_20074 | 0 | 1 | 0 | *Weissella* |
| **bASV_19224** | **0** | **1** | **0** | ***Weissella*** |
| **bASV_7915** | **0** | **1** | **0** | ***Bacillus*** |
| **bASV_16432** | **0** | **1** | **0** | ***Enterobacter*** |
| bASV_15224 | 0 | 1 | 0 | *Weissella* |
| bASV_490 | 0 | 1 | 0 | *Weissella* |
| bASV_23092 | 0 | 1 | 1 | *Leuconostoc* |
| **bASV_8941** | **0** | **1** | **0** | ***Pediococcus*** |
| **bASV_7220** | **0** | **1** | **0** | ***Weissella*** |
| bASV_7740 | 0 | 1 | 0 | *Pediococcus* |
| **bASV_20310** | **0** | **1** | **0** | ***Pediococcus*** |
| **bASV_12710** | **0** | **1** | **0** | ***Pediococcus*** |
| bASV_23809 | 0 | 1 | 0 | *Weissella* |
| bASV_14533 | 0 | 1 | 0 | *Leuconostoc* |
| **bASV_6386** | **0** | **1** | **0** | ***Weissella*** |
| **bASV_4327** | **0** | **1** | **0** | ***Weissella*** |
| bASV_6728 | 0 | 1 | 0 | unclassified |
| bASV_2411 | 0 | 1 | 0 | *Weissella* |
| **bASV_14344** | **0** | **1** | **0** | ***Lactobacillus*** |
| bASV_18472 | 0 | 1 | 0 | unclassified |
| bASV_10760 | 0 | 1 | 0 | *Weissella* |
| **bASV_6082** | **0** | **1** | **0** | ***Weissella*** |
| **bASV_16604** | **0** | **1** | **0** | ***Lactobacillus*** |
| **bASV_6702** | **0** | **1** | **0** | **unclassified** |
| bASV_15068 | 0 | 1 | 0 | *Weissella* |
| bASV_23545 | 0 | 1 | 0 | *Weissella* |
| bASV_5989 | 0 | 1 | 0 | *Leuconostoc* |
| **bASV_7433** | **0** | **1** | **0** | **unclassified** |
| **bASV_18855** | **0** | **1** | **0** | ***Lactobacillus*** |
| bASV_23674 | 0 | 1 | 0 | *Bacillus* |
| **bASV_469** | **0** | **1** | **0** | ***Pediococcus*** |
| **bASV_7156** | **0** | **1** | **0** | ***Weissella*** |
| **bASV_13799** | **0** | **1** | **0** | ***Bacillus*** |
| **bASV_18969** | **0** | **1** | **0** | ***Weissella*** |
| **bASV_15533** | **0** | **1** | **0** | ***Lactobacillus*** |
| bASV_15358 | 0 | 1 | 0 | *Weissella* |
| **bASV_6319** | **0** | **1** | **0** | ***Pediococcus*** |
| **bASV_14981** | **0** | **1** | **0** | **unclassified** |
| **bASV_20218** | **0** | **1** | **0** | ***Weissella*** |
| bASV_10149 | 0 | 1 | 0 | *Weissella* |
| **bASV_8380** | **0** | **1** | **0** | ***Bacillus*** |
| **bASV_3355** | **0** | **1** | **0** | ***Weissella*** |
| **bASV_11767** | **0** | **1** | **0** | ***Bacillus*** |
| **bASV_10678** | **0** | **1** | **0** | ***Weissella*** |
| **bASV_952** | **0** | **1** | **0** | ***Bacillus*** |
| **bASV_14452** | **0** | **1** | **0** | ***Weissella*** |
| **bASV_1660** | **0** | **1** | **0** | ***Weissella*** |
| **bASV_1455** | **0** | **1** | **0** | ***Weissella*** |
| **bASV_4698** | **0** | **1** | **0** | ***Weissella*** |
| **bASV_20987** | **0** | **1** | **0** | ***Weissella*** |
| **bASV_6542** | **0** | **1** | **0** | ***Weissella*** |
| **bASV_20747** | **0** | **1** | **0** | ***Weissella*** |
| **bASV_16088** | **0** | **1** | **0** | ***Lactobacillus*** |
| **bASV_23846** | **0** | **1** | **0** | ***Pediococcus*** |
| **bASV_16641** | **0** | **1** | **0** | **unclassified** |
| **bASV_10063** | **0** | **1** | **0** | **unclassified** |
| **bASV_4776** | **0** | **1** | **0** | ***Lactobacillus*** |
| **bASV_21011** | **0** | **1** | **0** | ***Lactobacillus*** |
| **bASV_10749** | **0** | **1** | **0** | ***Lactobacillus*** |
| **bASV_445** | **0** | **1** | **0** | ***Lactobacillus*** |
| **bASV_9953** | **0** | **1** | **0** | ***Weissella*** |
| **bASV_18728** | **0** | **0** | **1** | ***Leuconostoc*** |
| bASV_22918 | 0 | 0 | 1 | *Lactobacillus* |
| **bASV_18869** | **0** | **0** | **1** | ***Leuconostoc*** |
| **bASV_17441** | **0** | **0** | **1** | ***Lactobacillus*** |
| bASV_1766 | 0 | 0 | 1 | *Leuconostoc* |
| bASV_16946 | 0 | 0 | 1 | *Weissella* |
| **bASV_4108** | **0** | **0** | **1** | ***Leuconostoc*** |
| bASV_10059 | 0 | 0 | 1 | *Leuconostoc* |
| bASV_7272 | 0 | 0 | 1 | *Weissella* |
| **bASV_4941** | **0** | **0** | **1** | ***Weissella*** |
| **bASV_19308** | **0** | **0** | **1** | ***Lactobacillus*** |
| **bASV_22230** | **0** | **0** | **1** | ***Lactococcus*** |
| bASV_18746 | 0 | 0 | 1 | *Weissella* |
| bASV_16462 | 0 | 0 | 1 | *Lactobacillus* |
| bASV_19505 | 0 | 0 | 1 | *Weissella* |
| bASV_14803 | 0 | 0 | 1 | *Lactobacillus* |
| bASV_4174 | 0 | 0 | 1 | unclassified |
| **bASV_3253** | **0** | **0** | **1** | ***Lactobacillus*** |
| bASV_18719 | 0 | 0 | 1 | *Weissella* |
| **bASV_4100** | **0** | **0** | **1** | ***Lactobacillus*** |
| **bASV_11670** | **0** | **0** | **1** | ***Lactobacillus*** |
| **bASV_2428** | **0** | **0** | **1** | ***Lactococcus*** |
| **bASV_2521** | **0** | **0** | **1** | ***Klebsiella*** |
| **bASV_13403** | **0** | **0** | **1** | ***Lactobacillus*** |
| **bASV_14115** | **0** | **0** | **1** | ***Staphylococcus*** |
| **bASV_2198** | **0** | **0** | **1** | ***Lactobacillus*** |
| **bASV_11059** | **0** | **0** | **1** | ***Klebsiella*** |
| **bASV_15684** | **0** | **0** | **1** | ***Leuconostoc*** |
| **bASV_4447** | **0** | **0** | **1** | ***Lactobacillus*** |
| **bASV_8221** | **0** | **0** | **1** | ***Leuconostoc*** |
| bASV_21739 | 0 | 0 | 1 | *Lactobacillus* |
| **bASV_8493** | **0** | **0** | **1** | ***Providencia*** |
| **bASV_21454** | **0** | **0** | **1** | ***Lactobacillus*** |
| **bASV_10163** | **0** | **0** | **1** | ***Acinetobacter*** |
| **bASV_17156** | **0** | **0** | **1** | **unclassified** |
| bASV_16174 | 0 | 1 | 1 | *Weissella* |
| **bASV_23127** | **0** | **0** | **1** | ***Weissella*** |
| bASV_11212 | 0 | 0 | 1 | *Lactococcus* |
| **bASV_21013** | **0** | **0** | **1** | ***Lactobacillus*** |
| bASV_1012 | 0 | 0 | 1 | *Weissella* |
| **bASV_12590** | **0** | **0** | **1** | **unclassified** |
| **bASV_5783** | **0** | **0** | **1** | ***Klebsiella*** |
| **bASV_15948** | **0** | **0** | **1** | ***Weissella*** |
| **bASV_16799** | **0** | **0** | **1** | ***Leuconostoc*** |
| **bASV_22841** | **0** | **0** | **1** | ***Lactobacillus*** |
| bASV_16889 | 0 | 0 | 1 | *Staphylococcus* |
| **bASV_18352** | **0** | **0** | **1** | ***Providencia*** |
| **bASV_11634** | **0** | **0** | **1** | ***Leuconostoc*** |
| **bASV_1744** | **0** | **0** | **1** | ***Acinetobacter*** |
| **bASV_19514** | **0** | **0** | **1** | ***Leuconostoc*** |
| **bASV_5030** | **0** | **0** | **1** | ***Lactobacillus*** |
| **bASV_835** | **0** | **0** | **1** | ***Lactobacillus*** |
| **bASV_20796** | **0** | **0** | **1** | ***Weissella*** |
| **bASV_23568** | **0** | **0** | **1** | ***Leuconostoc*** |
| **bASV_18946** | **0** | **0** | **1** | ***Pediococcus*** |

**Note:** All ASVs in the table were obtained by indicator species analysis (*bi*ASV). Among them, the ASV marked in red was significantly related to fermentation parameters (*bp*ASV), while the ASVs shown in bold were not significantly correlated with the fermentation parameters but were significantly diverse in different MTD, and defined as bioturbation responding significant ASVs *(bs*ASV*)*.

**Supplementary Table 5** Definition of fungal ASV (*f*ASV) sensitive to bioturbation.

| Group | DD | CD | PD | Genus |
| --- | --- | --- | --- | --- |
| fASV_311 | 0 | 1 | 0 | *Hyphopichia* |
| fASV_259 | 0 | 1 | 1 | *Rhizopus* |
| fASV_418 | 0 | 1 | 0 | *Wickerhamomyces* |
| fASV_314 | 0 | 1 | 0 | *Hyphopichia* |
| **fASV_353** | **0** | **1** | **0** | ***Hyphopichia*** |
| **fASV_295** | **0** | **1** | **0** | **unclassified** |
| **fASV_586** | **0** | **1** | **0** | ***Hyphopichia*** |
| fASV_39 | 0 | 1 | 0 | *Thermoascus* |
| **fASV_126** | **0** | **1** | **0** | ***Candida*** |
| fASV_44 | 0 | 1 | 0 | *Thermoascus* |
| fASV_64 | 0 | 1 | 0 | *Paecilomyces* |
| fASV_436 | 0 | 0 | 1 | *Thermoascus* |
| fASV_460 | 0 | 0 | 1 | *Rhizomucor* |
| fASV_41 | 0 | 0 | 1 | *Rhizomucor* |
| fASV_284 | 0 | 0 | 1 | *Rhizomucor* |

**Note:** All ASVs in the table were obtained by indicator species analysis (*fi*ASV). Among them, the ASVs marked in red were significantly related to fermentation parameters (*fp*ASV), while the ASVs shown in bold were not significantly correlated with the fermentation parameters but were significantly diverse in different MTD, and defined as bioturbation responding significant ASVs *(bs*ASV*)*.

**Supplementary Table 6** Results of PERMANOVA testing of different type of MTD functional microbial communities.

| Type | Group | Bacteria | | Fungi | |
| --- | --- | --- | --- | --- | --- |
|  |  | R² | *p* | R² | *p* |
| LA | DD/PD | **0.189** | **0.026** | 0.104 | 0.179 |
|  | CD/PD | **0.325** | **0.009** | 0.165 | 0.147 |
|  | DD/CD | 0.052 | 0.355 | 0.015 | 0.739 |
|  | All | **0.227** | **0.01** | 0.11 | 0.144 |
| SA | DD/PD | **0.239** | **0.036** | **0.382** | **0.002** |
|  | CD/PD | **0.431** | **0.006** | **0.406** | **0.002** |
|  | DD/CD | 0.108 | 0.114 | 0.004 | 0.892 |
|  | All | **0.327** | **0.002** | **0.305** | **0.008** |
| EA | DD/PD | 0.029 | 0.723 | 0.085 | 0.264 |
|  | CD/PD | 0.083 | 0.513 | 0.077 | 0.264 |
|  | DD/CD | 0.023 | 0.723 | 0.067 | 0.264 |
|  | All | 0.057 | 0.539 | 0.099 | 0.202 |
| FA | DD/PD | 0.065 | 0.346 | 0.03 | 0.599 |
|  | CD/PD | 0.071 | 0.346 | 0.058 | 0.599 |
|  | DD/CD | 0.055 | 0.346 | 0.036 | 0.599 |
|  | All | 0.084 | 0.279 | 0.054 | 0.546 |

**Note:** LA, SA, EA and FA respectively represent liquefying, saccharifying, esterifying and fermenting activities

**Supplementary Table 7** Relative abundance of *e*ASVs (statistics to genus level) of different MTD.

| **Genus** | **LA** | | | **SA** | | |
| --- | --- | --- | --- | --- | --- | --- |
|  | **DD** | **CD** | **PD** | **DD** | **CD** | **PD** |
| ***Lactobacillus*** | **0.52ab** | **0.34b** | **0.74a** | **0.49ab** | **0.28b** | **0.66a** |
| ***Bacillus*** | **0.20ab** | **0.34a** | **0.02b** | 0.02a | 0.04a | 0.01a |
| *Enterobacter* | — | — | — | 0.00a | 0.07a | 0.00a |
| ***Lactococcus*** | **0.00b** | **0.00b** | **0.01a** | **0.00b** | **0.00b** | **0.01a** |
| ***Leuconostoc*** | **0.02b** | **0.04b** | **0.09a** | **0.03b** | **0.04b** | **0.09a** |
| *Pediococcus* | 0.02a | 0.03a | 0.01a | 0.01ab | 0.01a | 0.00b |
| *Staphylococcus* | 0.01a | 0.03a | 0.01a | 0.00a | 0.01a | 0.01a |
| *Streptomyces* | 0.04a | 0.03a | 0.00a | — | — | — |
| ***Weissella*** | **0.17a** | **0.16a** | **0.09b** | **0.44ab** | **0.53a** | **0.19b** |
| *Aspergillus* | 0.07a | 0.03a | 0.07a | — | — | — |
| *Candida* | 0.02a | 0.03a | 0.00a | 0.04a | 0.07a | 0.00a |
| ***Hyphopichia*** | **—** | **—** | **—** | **0.36a** | **0.38a** | **0.01b** |
| *Rasamsonia* | 0.02a | 0.00a | 0.00a | — | — | — |
| ***Rhizomucor*** | **—** | **—** | **—** | **0.51b** | **0.46b** | **0.98a** |
| *Thermoascus* | 0.70a | 0.64a | 0.93a | 0.00a | 0.00a | 0.00a |
| *Thermomyces* | 0.00a | 0.00a | 0.00a | — | — | — |
| ***Wickerhamomyces*** | 0.11a | 0.22a | 0.00a | **0.04a** | **0.03ab** | **0.00b** |
| **Genus** | **EA** | | | **FA** | | |
|  | **DD** | **CD** | **PD** | **DD** | **CD** | **PD** |
| *Lactobacillus* | 0.10a | 0.07a | 0.15a | 0.09a | 0.07a | 0.09a |
| *Bacillus* | 0.22a | 0.34a | 0.12a | 0.01a | 0.03a | 0.01a |
| ***Leuconostoc*** | 0.01a | 0.00a | 0.03a | **0.05b** | **0.09ab** | **0.19a** |
| ***Pediococcus*** | 0.09a | 0.14a | 0.11a | **0.03ab** | **0.06a** | **0.02b** |
| *Staphylococcus* | 0.12a | 0.05a | 0.16a | 0.16a | 0.10a | 0.18a |
| *Streptomyces* | 0.04a | 0.03a | 0.00a | 0.02a | 0.03a | 0.02a |
| *Thermoactinomyces* | 0.01a | 0.03a | 0.01a | — | — | — |
| *Weissella* | 0.39a | 0.33a | 0.39a | 0.63a | 0.55a | 0.46a |
| *Aspergillus* | 0.01a | 0.02a | 0.00a | 0.00a | 0.00a | 0.00a |
| *Candida* | 0.00a | 0.03a | 0.00a | 0.01a | 0.11a | 0.00a |
| ***Hyphopichia*** | **0.03ab** | **0.11a** | **0.00b** | 0.03a | 0.06a | 0.01a |
| *Pichia* | 0.48a | 0.33a | 0.35a | 0.07a | 0.05a | 0.13a |
| *Rhizopus* | 0.04a | 0.09a | 0.01a | 0.21a | 0.19a | 0.10a |
| *Thermoascus* | 0.27a | 0.38a | 0.53a | 0.46a | 0.50a | 0.63a |
| *Thermomyces* | 0.16a | 0.04a | 0.12a | 0.20a | 0.04a | 0.12a |
| *Wickerhamomyces* | — | — | — | 0.01a | 0.03a | 0.00a |

**Note:** Values in the same row with different letters indicate significant difference (*P* < 0.05) according to ANOVA test. LA, SA, EA and FA respectively represent liquefying, saccharifying, esterifying and fermenting activities.
